# Draft Genome Sequence of Gephyronic Acid Producer *Cystobacter violaceus* Strain Cb vi76

**DOI:** 10.1128/genomeA.01299-14

**Published:** 2014-12-11

**Authors:** D. Cole Stevens, Jeanette Young, Rory Carmichael, John Tan, Richard E. Taylor

**Affiliations:** a Department of Chemistry and Biochemistry, University of Notre Dame, Notre Dame, Indiana, USA; b Genomics and Bioinformatics Core Facility, University of Notre Dame, Notre Dame, Indiana, USA

## Abstract

A draft genome sequence of *Cystobacter violaceus* strain Cb vi76, which produces the eukaryotic protein synthesis inhibitor gephyronic acid, has been obtained. The genome contains numerous predicted secondary metabolite clusters, including the gephyronic acid biosynthetic pathway. This genome will contribute to the investigation of secondary metabolism in other *Cystobacter* strains.

## GENOME ANNOUNCEMENT

Myxobacteria are an excellent source of structurally diverse, bioactive natural products (1, 2). Isolated from a soil sample with plant residues near Mitgamr, Egypt, in 2007, *Cystobacter violaceus* strain Cb vi76, DSM 14727, produces the cytotoxic polyketide natural product gephyronic acid, which is a potent and selective inhibitor of eukaryotic protein synthesis (3–6). Herein we present a draft genome sequence of the strain collected in our efforts to determine the biosynthetic pathway for gephyronic acid.

*C. violaceus* genomic DNA was sequenced using a Roche 454 GS FLX sequencer with a combination of shotgun sequencing and 3-kb paired-end sequencing at the Genomics and Bioinformatics Core Facility at the University of Notre Dame. Shotgun Titanium sequencing yielded 552,177 reads, which were assembled using the Roche Newbler Assembler version 2.3 into 431 contigs comprising 12,505,879 bp in total (13× coverage). An additional 2× coverage was supplied utilizing paired-end sequences that were mapped to the shotgun genome sequence to fill gaps and orient contigs. Based on this assembly, we generated the *C. violaceus* Cb vi76 draft genome sequence consisting of 12,570,057 bp distributed among 83 scaffolds with a GC content of 68.9%, respectively totaling an approximate 15× coverage of the genome.

The *C. violaceus* genome was analyzed and annotated using RAST version 4.0 (7) and the NCBI Prokaryotic Genomes Annotation Pipeline (PGAP) (http://www.ncbi.nlm.nih.gov/genome/annotation_prok/). Analysis of the unclosed draft genome sequence for *C. violaceus* provided an estimated genome size of 12.57 Mbp. The 431 contigs contain 8,201 putative coding sequences (CDS). Genes are evenly distributed between the forward (51.2%) and reverse (48.8%) strands. The average length of the CDSs is 1,065 bp, and 50.1% of the CDSs encode proteins whose functions are unknown. In addition to the identified protein functionalities, single 5S, 16S, and 23S rRNA genes were annotated utilizing RNAmmer (8). The search server, tRNAscan-SE, annotated 79 tRNA genes representing all 20 common amino acids (9).

Additional genome analysis with antiSMASH version 1.1.0 predicted several secondary metabolite biosynthetic gene clusters, including biosynthetic pathways for 5 lantibiotics, 2 bacteriocins, 6 terpenes, 4 polyketides, 7 nonribosomal peptides, and 8 polyketide/nonribosomal peptide hybrids (10, 11). All predicted secondary metabolite gene clusters for polyketide synthase and/or nonribosomal peptide synthetase are of unknown structure, except the gephyronic acid (6). Further inspection of the proposed gene clusters revealed that several are in fact gene cluster fragments, befitting of the draft quality of the *C. violaceus* genome sequence.

The breadth of proposed biosynthetic enzymes with unknown cognate natural products as well as the known gephyronic acid biosynthetic pathway exemplify the potential access to natural products intrinsic to myxobacteria such as *C. violaceus*. We believe the draft genome sequence will help facilitate future work for further investigation of secondary metabolism in myxobacteria.

### Nucleotide sequence accession numbers.

This whole-genome shotgun project has been deposited in DDBJ/ENA/GenBank under the accession number JPMI00000000. The version described in this paper is the ﬁrst version, JPMI01000000.

## References

[1] Wenzel SC Müller R 2009 The impact of genomics on the exploitation of the myxobacterial secondary metabolome. Nat. Prod. Rep. 26:1385–1407. 10.1039/b817073h. 19844638

[2] Schäberle TF Lohr F Schmitz A König GM 2014 Antibiotics from myxobacteria. Nat. Prod. Rep. 31:953–972. 10.1039/c4np00011k. 24841474

[3] Sasse F Steinmetz H Höfle G Reichenbach H 1995 Gephyronic acid, a novel inhibitor of eukaryotic protein synthesis from *Archangium gephyra* (myxobacteria): production, isolation, physico-chemical and biological properties, and mechanism of action. J. Antibiot. 48:21–25. 10.7164/antibiotics.48.21. 7868385

[4] Nicolas L Anderl T Sasse F Steinmetz H Jansen R Höfle G Laschat S Taylor RE 2011 Gephyronic acid, a missing link between polyketide inhibitors of eukaryotic protein synthesis (part I): structural revision and stereochemical assignment of gephyronic acid. Angew. Chem. Int. Ed. 50:938–941. 10.1002/anie.201005530. 21246696

[5] Anderl T Nicolas L Münkemer J Baro A Sasse F Steinmetz H Jansen R Höfle G Taylor RE Laschat S 2011 Gephyronic acid, a missing link between polyketide inhibitors of eukaryotic protein synthesis (part II): total synthesis of gephyronic acid. Angew. Chem. Int. Ed. 50:942–955. 10.1002/anie.201005605. 21246697

[6] Young J Stevens DC Carmichael R Tan J Rachid S Boddy CN Müller R Taylor RE 2013 Elucidation of gephyronic acid biosynthetic pathway revealed unexpected SAM-dependent methylations. J. Nat. Prod. 76:2269–2276. 10.1021/np400629v. 24298873

[7] Aziz RK Bartels D Best AA DeJongh M Disz T Edwards RA Formsma K Gerdes S Glass EM Kubal M Meyer F Olsen GJ Olson R Osterman AL Overbeek RA McNeil LK Paarmann D Paczian T Parrello B Pusch GD Reich C Stevens R Vassieva O Vonstein V Wilke A Zagnitko O 2008 The RAST server: rapid annotations using subsystems technology. BMC Genomics 9:75. 10.1186/1471-2164-9-75. 18261238 PMC2265698

[8] Lagesen K Hallin P Rødland EA Staerfeldt HH Rognes T Ussery DW 2007 RNAmmer: consistent and rapid annotation of ribosomal RNA genes. Nucleic Acids Res. 35:3100–3108. 10.1093/nar/gkm160. 17452365 PMC1888812

[9] Lowe TM Eddy SR 1997 tRNAscan-SE: A program for improved detection of transfer RNA genes in genomic sequence. Nucleic Acids Res. 25:955–964. 10.1093/nar/25.5.0955. 9023104 PMC146525

[10] Medema MH Blin K Cimermancic P de Jager V Zakrzewski P Fischbach MA Weber T Takano E Breitling R 2011 antiSMASH: rapid identification, annotation and analysis of secondary metabolite biosynthesis gene clusters in bacterial and fungal genome sequences. Nucleic Acids Res. 39:W339–W346. 10.1093/nar/gkr466. 21672958 PMC3125804

[11] Boddy CN 2014 Bioinformatics tools for genome mining of polyketide and non-ribosomal peptides. J. Ind. Microbiol. Biotechnol. 41:443–450. 10.1007/s10295-013-1368-1. 24174214

